# Crystal structure of ethyl 6-methyl-2-sulfanyl­idene-4-(thio­phen-2-yl)-1,2,3,4-tetra­hydro­pyrimidine-5-carboxyl­ate

**DOI:** 10.1107/S2056989014027741

**Published:** 2015-01-03

**Authors:** M. Suresh, M. Syed Ali Padusha, J. Josephine Novina, G. Vasuki, Vijayan Viswanathan, Devadasan Velmurugan

**Affiliations:** a PG & Research Department of Chemistry, Jamal Mohamed College (Autonomous), Tiruchirappalli-20, India; b Department of Physics, Idhaya College for Women, Kumbakonam-1, India; c Department of Physics, Kunthavai Naachiar Government Arts College (W) (Autonomous), Thanjavur-7, India; d Centre of Advanced study in Crystallography and Biophysics, University of Madras, Guindy Campus, Chennai-25, India

**Keywords:** crystal structure, pyrimidine, hydrogen bonding, C—H⋯π inter­actions, conformation

## Abstract

In the title compound, C_12_H_14_N_2_O_2_S_2_, the di­hydro­pyrimidine ring adopts a sofa conformation, with the C atom bearing the thienyl ring lying above the plane of the five remaining approximately coplanar (r.m.s. deviation = 0.0405 Å) atoms of the ring. The dihedral angle between the five near coplanar atoms of the ring and the thienyl ring is 89.78 (11)°. In the crystal, mol­ecules are linked into a supra­molecular chain along [100] *via* N—H⋯O(carbon­yl) hydrogen bonds. Inversion-related chains are linked into double chains *via* N—H⋯S(thione) hydrogen bonds. The three-dimensional architecture also features meth­yl–thienyl C—H⋯π inter­actions.

## Related literature   

For general background and the biological activity of di­hydro­pyrimidino­nes, see: Phucho *et al.* (2009 ▸); Patil *et al.* (2011 ▸).
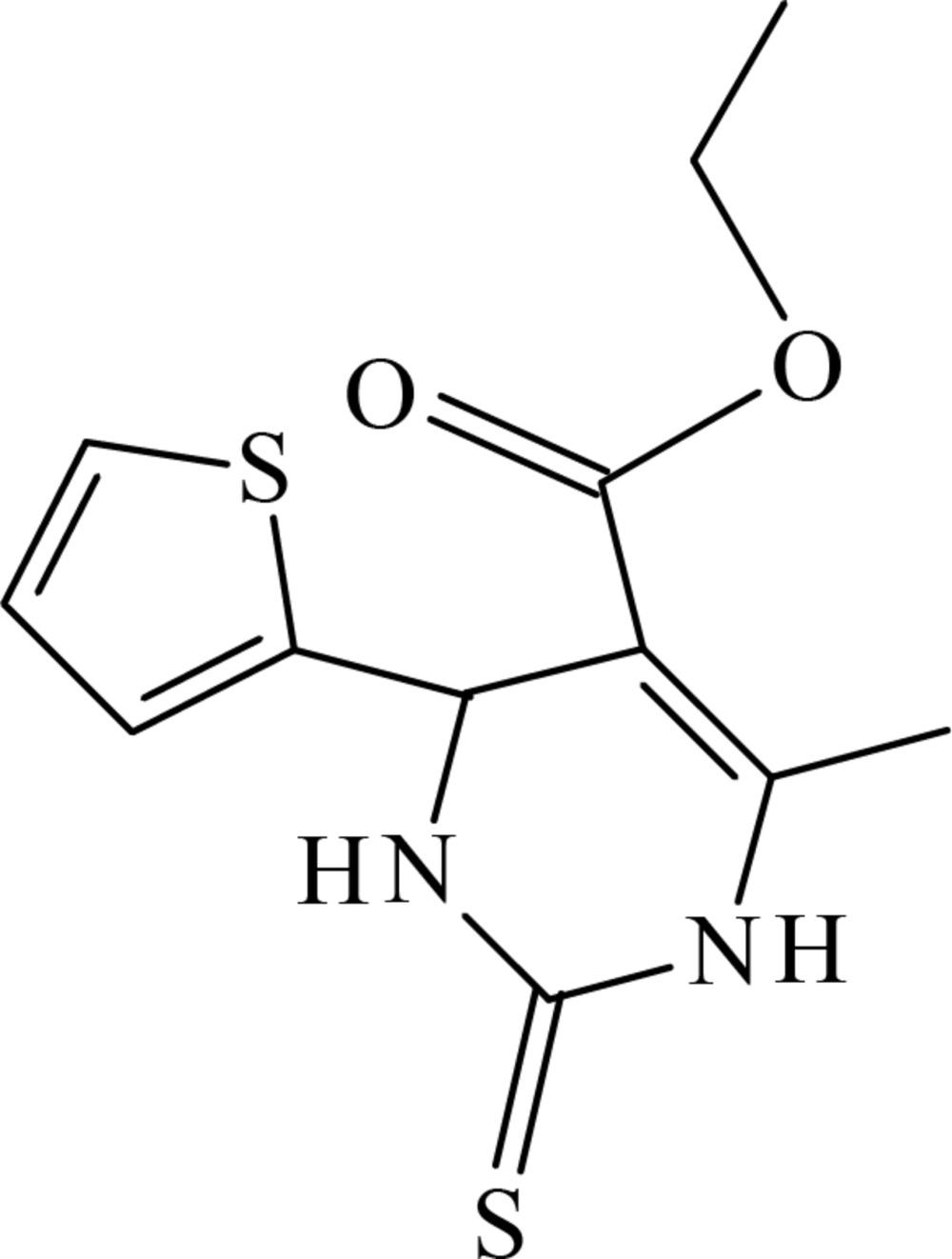


## Experimental   

### Crystal data  

C_12_H_14_N_2_O_2_S_2_
*M* *_r_* = 282.37
Triclinic, 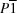

*a* = 7.3069 (1) Å
*b* = 8.3267 (1) Å
*c* = 11.2461 (1) Å
α = 90.109 (1)°
β = 95.156 (1)°
γ = 101.276 (1)°
*V* = 668.18 (1) Å^3^
*Z* = 2
Mo *K*α radiation
μ = 0.39 mm^−1^
*T* = 293 K
0.20 × 0.15 × 0.10 mm

### Data collection  

Bruker APEXII CCD diffractometer
Absorption correction: multi-scan (*SADABS*; Bruker, 2008 ▸) *T*_min_ = 0.925, *T*_max_ = 0.962
10188 measured reflections
2762 independent reflections
2325 reflections with *I* > 2σ(*I*)
*R*_int_ = 0.021

### Refinement  

*R*[*F* ^2^ > 2σ(*F* ^2^)] = 0.060
*wR*(*F* ^2^) = 0.189
*S* = 1.06
2762 reflections
165 parameters
H-atom parameters constrained
Δρ_max_ = 0.64 e Å^−3^
Δρ_min_ = −0.40 e Å^−3^

### 

Data collection: *APEX2* (Bruker, 2008 ▸); cell refinement: *APEX2* and *SAINT* (Bruker, 2008 ▸); data reduction: *SAINT* and *XPREP* (Bruker, 2008 ▸); program(s) used to solve structure: *SIR92* (Altomare *et al.*, 1993 ▸); program(s) used to refine structure: *SHELXL97* (Sheldrick, 2008 ▸); molecular graphics: *ORTEP-3 for Windows* (Farrugia, 2012 ▸) and *Mercury* (Macrae *et al.*, 2008 ▸); software used to prepare material for publication: *PLATON* (Spek, 2009 ▸).

## Supplementary Material

Crystal structure: contains datablock(s) I, global. DOI: 10.1107/S2056989014027741/tk5352sup1.cif

Structure factors: contains datablock(s) I. DOI: 10.1107/S2056989014027741/tk5352Isup2.hkl

Supporting information file. DOI: 10.1107/S2056989014027741/tk5352Isup3.cml

**Fig. 1.**
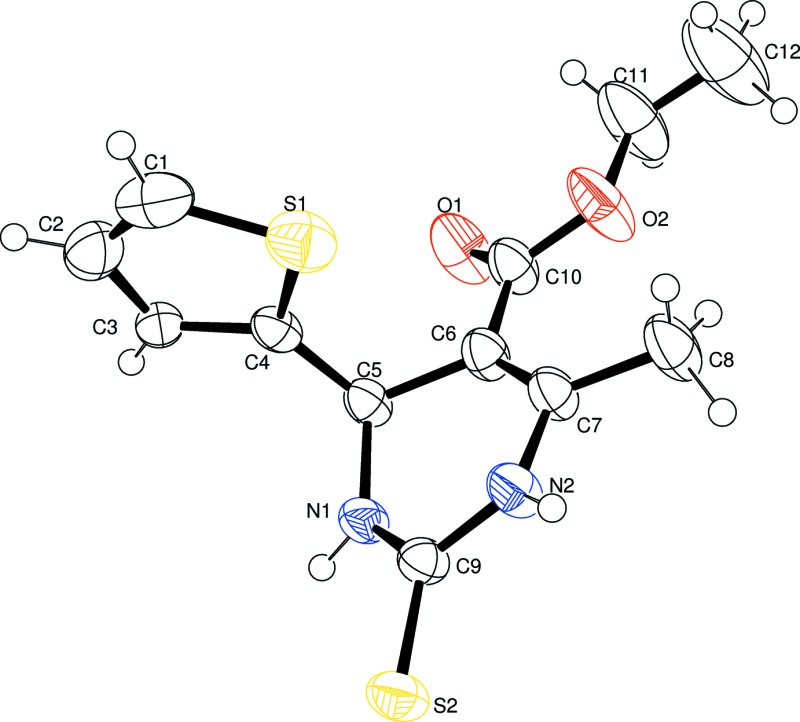
The molecular structure of the title compound, with the atom labelling. Displacement ellipsoids are drawn at the 50% probability level.

**Fig. 2.**
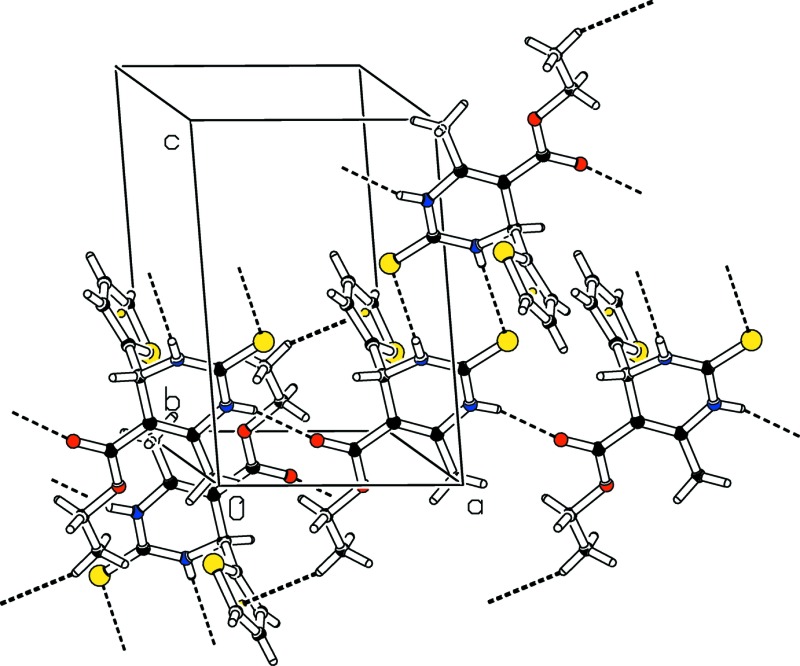
Partial crystal packing of the title compound, showing the R22(8) ring motif, viewed along the b axis. Hydrogen bonds are shown as dashed lines.

**Fig. 3.**
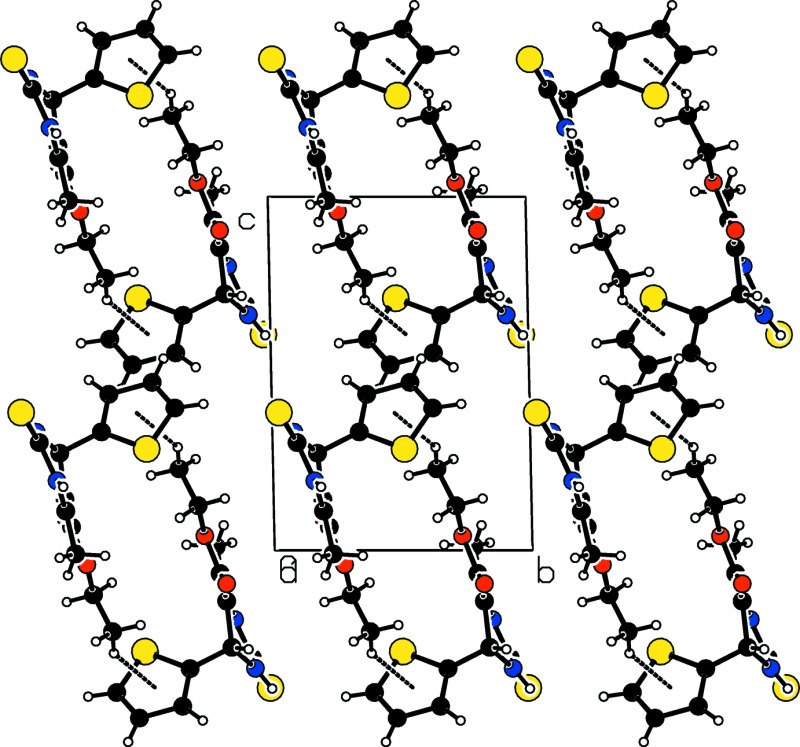
Part of the crystal packing of the title compound, showing C—H···π interactions. Viewed along the a axis.

. DOI: 10.1107/S2056989014027741/tk5352fig1.tif
The mol­ecular structure of the title compound, with the atom labelling. Displacement ellipsoids are drawn at the 50% probability level.

b . DOI: 10.1107/S2056989014027741/tk5352fig2.tif
Partial crystal packing of the title compound, showing the 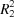
(8) ring motif, viewed along the *b* axis. Hydrogen bonds are shown as dashed lines.

a . DOI: 10.1107/S2056989014027741/tk5352fig3.tif
Part of the crystal packing of the title compound, showing C—H⋯π inter­actions. Viewed along the *a* axis.

**Fig. 4.**
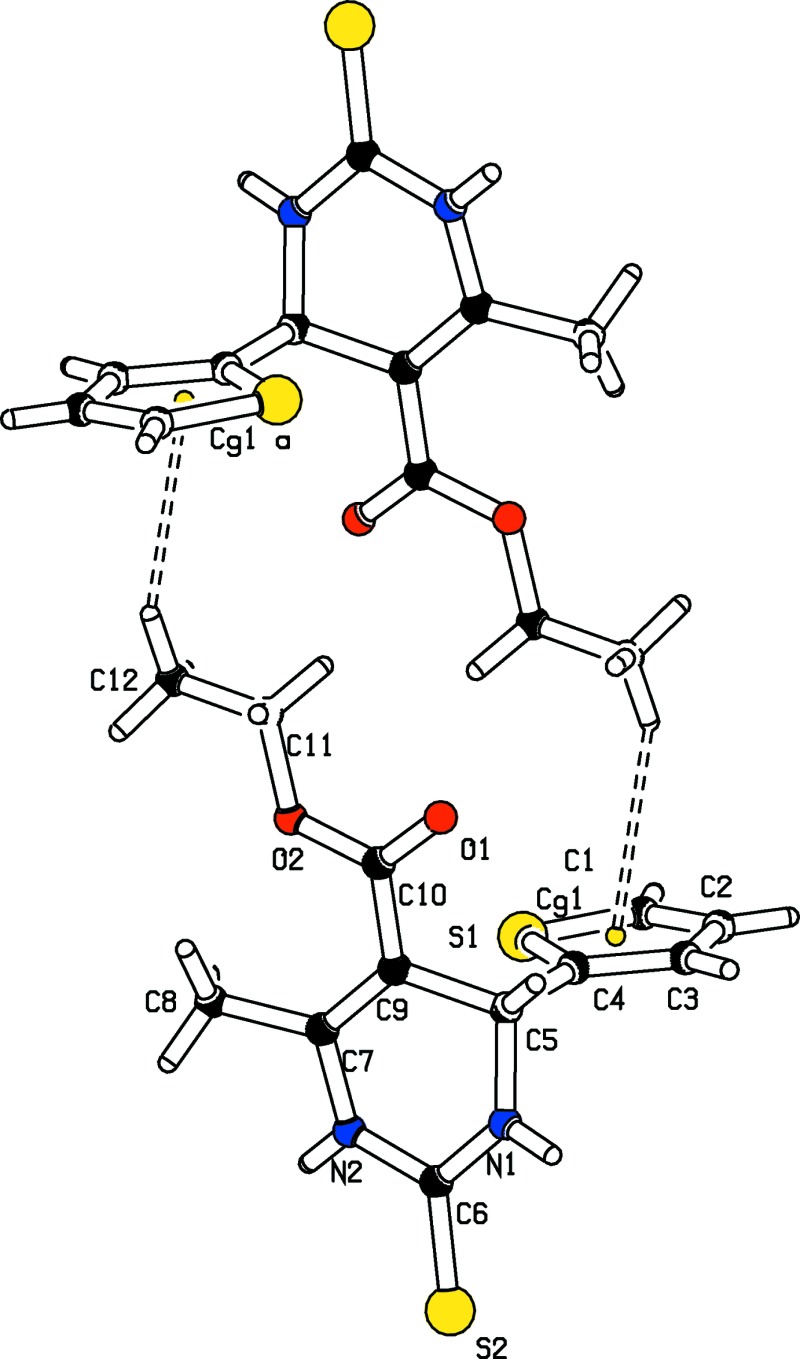
Image showing the C—H···π interactions.

. DOI: 10.1107/S2056989014027741/tk5352fig4.tif
Image showing the C—H⋯π inter­actions.

CCDC reference: 1040426

Additional supporting information:  crystallographic information; 3D view; checkCIF report

## Figures and Tables

**Table 1:** Hydrogen-bond geometry (, ) *Cg*1 is the centroid of the S1/C1C4 thiophene ring.

*D*H*A*	*D*H	H*A*	*D* *A*	*D*H*A*
N1H1*N*1S2^i^	0.86	2.63	3.408(2)	151
N2H2*N*2O1^ii^	0.86	2.15	2.984(3)	162
C12H12*B* *Cg*1^iii^	0.96	2.81	3.664(6)	149

Symmetry codes: (i) 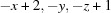; (ii) 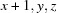; (iii) 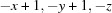.

## supplementary crystallographic information

### S1. Structural commentary

Pyrimidino­nes or di­hydro­pyrimidino­nes (DHPMs) are well known for their wide range of bioactivities and their applications in the field of drug research have stimulated the invention of a wide range of synthetic methods for their preparation and chemical transformations (Phucho *et al.*, 2009). Several functionalized di­hydro­pyrimidino­nes are used as calcium channel modulators, Ca-antagonists, vasodilative and anti-hypertensive agents (Patil *et al.*, 2011). Against this background and in order to obtain detailed information on its molecular conformation, the structure of the title compound has been determined and the results are presented herein.

The asymmetric unit of the title compound is illustrated in Fig. 1. The di­hydro­pyrimidine ring adopts a sofa conformation, with puckering parameters q2 = 0.283 Å, q3 = -0.102 Å, Q = 0.301 Å, Θ = 109.8° and Φ = 232.1°. The dihedral angle between the mean plane of the five essentially planar atoms (N1/C9/N2/C7/C6) of the di­hydro­pyrimidine ring [maximum deviation 0.1944 (18) Å for C5] and the thio­phene ring (C1—C4/S1) is 89.78 (11)°.

In the crystal, the molecules are linked *via* a pair of N—H···S hydrogen bonds forming an inversion dimers with *R*^2^_2_(8) ring motif and form a chain parallel to the *bc* plane *via* N—H···O hydrogen bonds (Fig. 2). Additional stabilization to the structure is afforded by C—H···π contacts (Table 1, Figs 3 and 4).

### S2. Synthesis and crystallization

A mixture of ethyl aceto­acetate (0.13 ml, 0.001 mol), thio­phene-2-carboxaldehyde (0.1 ml, 0.001 mol) and thio­urea (0.228 g, 0.003 mol) in ethanol (5 ml) was heated under reflux in the presence of cerium chloride heptahydrate (25%) for 1 h (monitored by TLC). After the completion of the reaction, the reaction mixture was cooled to room temperature and poured onto crushed ice and stirred for 5–10 min. The solid was separated and filtered under suction, washed with ice-cold water (50 ml), and then recrystallized from hot ethanol to afford pure product [m.pt: 421 K; yield: 98%].

### S3. Refinement

H atoms were placed in geometrically idealized positions and constrained to ride on their parent atoms with C—H distances fixed in the range 0.93–0.98 Å and N—H = 0.86 Å with *U*_iso_(H) = 1.5*U*_eq_(CH_3_) and 1.2*U*_eq_(CH_2_,CH, NH).

### Crystal data

**Table:**

C_12_H_14_N_2_O_2_S_2_	*Z* = 2
*M**_r_* = 282.37	*F*(000) = 296
Triclinic, *P*1	*D*_x_ = 1.403 Mg m^−^^3^
Hall symbol: -P 1	Mo *K*α radiation, λ = 0.71073 Å
*a* = 7.3069 (1) Å	Cell parameters from 2762 reflections
*b* = 8.3267 (1) Å	θ = 1.8–26.5°
*c* = 11.2461 (1) Å	µ = 0.39 mm^−^^1^
α = 90.109 (1)°	*T* = 293 K
β = 95.156 (1)°	Block, colourless
γ = 101.276 (1)°	0.20 × 0.15 × 0.10 mm
*V* = 668.18 (1) Å^3^	

### Data collection

**Table:**

Bruker APEXII CCD diffractometer	2762 independent reflections
Radiation source: fine-focus sealed tube	2325 reflections with *I* > 2σ(*I*)
Graphite monochromator	*R*_int_ = 0.021
ω and φ scan	θ_max_ = 26.5°, θ_min_ = 1.8°
Absorption correction: multi-scan (*SADABS*; Bruker, 2008)	*h* = −9→9
*T*_min_ = 0.925, *T*_max_ = 0.962	*k* = −10→10
10188 measured reflections	*l* = −14→14

### Refinement

**Table:**

Refinement on *F*^2^	Primary atom site location: structure-invariant direct methods
Least-squares matrix: full	Secondary atom site location: difference Fourier map
*R*[*F*^2^ > 2σ(*F*^2^)] = 0.060	Hydrogen site location: inferred from neighbouring sites
*wR*(*F*^2^) = 0.189	H-atom parameters constrained
*S* = 1.06	*w* = 1/[σ^2^(*F*_o_^2^) + (0.1181*P*)^2^ + 0.5067*P*] where *P* = (*F*_o_^2^ + 2*F*_c_^2^)/3
2762 reflections	(Δ/σ)_max_ < 0.001
165 parameters	Δρ_max_ = 0.64 e Å^−^^3^
0 restraints	Δρ_min_ = −0.40 e Å^−^^3^

### Special details

**Table:**

Geometry. All e.s.d.'s (except the e.s.d. in the dihedral angle between two l.s. planes) are estimated using the full covariance matrix. The cell e.s.d.'s are taken into account individually in the estimation of e.s.d.'s in distances, angles and torsion angles; correlations between e.s.d.'s in cell parameters are only used when they are defined by crystal symmetry. An approximate (isotropic) treatment of cell e.s.d.'s is used for estimating e.s.d.'s involving l.s. planes.
Refinement. Refinement of *F*^2^ against ALL reflections. The weighted *R*-factor *wR* and goodness of fit *S* are based on *F*^2^, conventional *R*-factors *R* are based on *F*, with *F* set to zero for negative *F*^2^. The threshold expression of *F*^2^ > σ(*F*^2^) is used only for calculating *R*-factors(gt) *etc*. and is not relevant to the choice of reflections for refinement. *R*-factors based on *F*^2^ are statistically about twice as large as those based on *F*, and *R*- factors based on ALL data will be even larger.

### Fractional atomic coordinates and isotropic or equivalent isotropic displacement parameters (Å^2^)

**Table:**

	*x*	*y*	*z*	*U*_iso_*/*U*_eq_	
C1	0.8629 (5)	0.6238 (4)	0.4026 (3)	0.0584 (9)	
H1	0.9131	0.7349	0.4141	0.070*	
C2	0.7502 (5)	0.5376 (4)	0.4750 (3)	0.0534 (8)	
H2	0.7143	0.5833	0.5425	0.064*	
C3	0.6877 (4)	0.3688 (3)	0.4414 (2)	0.0341 (5)	
H3	0.6069	0.2922	0.4819	0.041*	
C4	0.7703 (3)	0.3372 (3)	0.3350 (2)	0.0338 (5)	
C5	0.7577 (3)	0.1734 (3)	0.2747 (2)	0.0336 (6)	
H5	0.6314	0.1088	0.2803	0.040*	
C9	1.0680 (4)	0.0949 (3)	0.3047 (2)	0.0335 (5)	
C7	0.9646 (4)	0.1925 (4)	0.1110 (2)	0.0384 (6)	
C8	1.0343 (5)	0.2269 (5)	−0.0103 (3)	0.0581 (9)	
H8A	0.9320	0.1960	−0.0709	0.087*	
H8B	1.1295	0.1651	−0.0216	0.087*	
H8C	1.0856	0.3417	−0.0157	0.087*	
C6	0.7909 (4)	0.1904 (4)	0.1437 (2)	0.0371 (6)	
C10	0.6280 (4)	0.2135 (4)	0.0657 (2)	0.0439 (7)	
C11	0.5131 (5)	0.2937 (7)	−0.1262 (3)	0.0817 (14)	
H11A	0.4303	0.1894	−0.1465	0.098*	
H11B	0.4413	0.3655	−0.0918	0.098*	
C12	0.5900 (7)	0.3646 (9)	−0.2304 (4)	0.115 (2)	
H12A	0.6581	0.4738	−0.2117	0.172*	
H12B	0.4906	0.3682	−0.2916	0.172*	
H12C	0.6732	0.2996	−0.2582	0.172*	
N1	0.8939 (3)	0.0838 (3)	0.33419 (19)	0.0359 (5)	
H1N1	0.8586	0.0204	0.3916	0.043*	
N2	1.1028 (3)	0.1610 (3)	0.1964 (2)	0.0402 (5)	
H2N2	1.2174	0.1848	0.1796	0.048*	
O1	0.4710 (3)	0.1889 (4)	0.0948 (2)	0.0673 (8)	
O2	0.6667 (3)	0.2699 (4)	−0.0409 (2)	0.0718 (9)	
S1	0.90646 (12)	0.51126 (11)	0.28681 (8)	0.0557 (3)	
S2	1.23669 (10)	0.02700 (10)	0.38956 (6)	0.0442 (3)	

### Atomic displacement parameters (Å^2^)

**Table:**

	*U*^11^	*U*^22^	*U*^33^	*U*^12^	*U*^13^	*U*^23^
C1	0.0543 (19)	0.0410 (17)	0.077 (2)	0.0109 (14)	−0.0128 (17)	0.0012 (16)
C2	0.0529 (19)	0.059 (2)	0.0520 (17)	0.0243 (15)	−0.0017 (14)	−0.0055 (15)
C3	0.0345 (13)	0.0374 (13)	0.0329 (12)	0.0136 (10)	0.0015 (10)	0.0007 (10)
C4	0.0287 (12)	0.0411 (14)	0.0324 (12)	0.0089 (10)	0.0026 (9)	0.0086 (10)
C5	0.0271 (12)	0.0441 (14)	0.0303 (12)	0.0075 (10)	0.0045 (9)	0.0077 (10)
C9	0.0328 (13)	0.0345 (13)	0.0340 (12)	0.0074 (10)	0.0051 (10)	0.0073 (10)
C7	0.0320 (13)	0.0538 (16)	0.0308 (12)	0.0105 (11)	0.0058 (10)	0.0089 (11)
C8	0.0421 (16)	0.101 (3)	0.0375 (15)	0.0257 (17)	0.0135 (12)	0.0224 (16)
C6	0.0310 (13)	0.0497 (16)	0.0311 (13)	0.0083 (11)	0.0044 (10)	0.0047 (11)
C10	0.0327 (14)	0.0662 (19)	0.0343 (13)	0.0121 (12)	0.0058 (11)	0.0096 (12)
C11	0.0403 (18)	0.156 (5)	0.0490 (19)	0.023 (2)	−0.0035 (15)	0.034 (2)
C12	0.061 (3)	0.214 (7)	0.077 (3)	0.041 (3)	0.010 (2)	0.066 (4)
N1	0.0350 (11)	0.0430 (12)	0.0333 (11)	0.0128 (9)	0.0101 (9)	0.0120 (9)
N2	0.0272 (11)	0.0596 (15)	0.0360 (11)	0.0117 (10)	0.0077 (9)	0.0138 (10)
O1	0.0319 (11)	0.126 (2)	0.0474 (12)	0.0213 (13)	0.0070 (9)	0.0226 (14)
O2	0.0369 (12)	0.138 (3)	0.0425 (12)	0.0217 (14)	0.0055 (9)	0.0377 (14)
S1	0.0519 (5)	0.0531 (5)	0.0613 (5)	0.0053 (4)	0.0108 (4)	0.0143 (4)
S2	0.0372 (4)	0.0567 (5)	0.0429 (4)	0.0181 (3)	0.0060 (3)	0.0170 (3)

### Geometric parameters (Å, º)

**Table:**

C1—C2	1.322 (5)	C7—C8	1.508 (4)
C1—S1	1.691 (4)	C8—H8A	0.9600
C1—H1	0.9300	C8—H8B	0.9600
C2—C3	1.429 (4)	C8—H8C	0.9600
C2—H2	0.9300	C6—C10	1.458 (4)
C3—C4	1.434 (3)	C10—O1	1.200 (3)
C3—H3	0.9300	C10—O2	1.323 (3)
C4—C5	1.505 (4)	C11—C12	1.425 (6)
C4—S1	1.709 (3)	C11—O2	1.454 (4)
C5—N1	1.472 (3)	C11—H11A	0.9700
C5—C6	1.516 (3)	C11—H11B	0.9700
C5—H5	0.9800	C12—H12A	0.9600
C9—N1	1.329 (3)	C12—H12B	0.9600
C9—N2	1.363 (3)	C12—H12C	0.9600
C9—S2	1.677 (3)	N1—H1N1	0.8600
C7—C6	1.350 (4)	N2—H2N2	0.8600
C7—N2	1.393 (3)		
			
C2—C1—S1	113.1 (3)	H8A—C8—H8C	109.5
C2—C1—H1	123.5	H8B—C8—H8C	109.5
S1—C1—H1	123.5	C7—C6—C10	126.4 (2)
C1—C2—C3	114.9 (3)	C7—C6—C5	119.0 (2)
C1—C2—H2	122.6	C10—C6—C5	114.5 (2)
C3—C2—H2	122.6	O1—C10—O2	121.5 (3)
C2—C3—C4	108.7 (3)	O1—C10—C6	124.0 (3)
C2—C3—H3	125.7	O2—C10—C6	114.5 (2)
C4—C3—H3	125.7	C12—C11—O2	108.3 (3)
C3—C4—C5	126.9 (2)	C12—C11—H11A	110.0
C3—C4—S1	111.2 (2)	O2—C11—H11A	110.0
C5—C4—S1	121.73 (19)	C12—C11—H11B	110.0
N1—C5—C4	110.9 (2)	O2—C11—H11B	110.0
N1—C5—C6	109.0 (2)	H11A—C11—H11B	108.4
C4—C5—C6	111.9 (2)	C11—C12—H12A	109.5
N1—C5—H5	108.3	C11—C12—H12B	109.5
C4—C5—H5	108.3	H12A—C12—H12B	109.5
C6—C5—H5	108.3	C11—C12—H12C	109.5
N1—C9—N2	115.7 (2)	H12A—C12—H12C	109.5
N1—C9—S2	123.97 (19)	H12B—C12—H12C	109.5
N2—C9—S2	120.33 (19)	C9—N1—C5	124.1 (2)
C6—C7—N2	119.0 (2)	C9—N1—H1N1	118.0
C6—C7—C8	128.0 (2)	C5—N1—H1N1	118.0
N2—C7—C8	113.1 (2)	C9—N2—C7	124.1 (2)
C7—C8—H8A	109.5	C9—N2—H2N2	117.9
C7—C8—H8B	109.5	C7—N2—H2N2	117.9
H8A—C8—H8B	109.5	C10—O2—C11	118.6 (2)
C7—C8—H8C	109.5	C1—S1—C4	92.16 (16)
			
S1—C1—C2—C3	−0.2 (4)	C5—C6—C10—O1	−14.5 (5)
C1—C2—C3—C4	1.0 (4)	C7—C6—C10—O2	−12.6 (5)
C2—C3—C4—C5	174.0 (2)	C5—C6—C10—O2	163.4 (3)
C2—C3—C4—S1	−1.3 (3)	N2—C9—N1—C5	−16.3 (4)
C3—C4—C5—N1	−81.0 (3)	S2—C9—N1—C5	166.1 (2)
S1—C4—C5—N1	93.8 (2)	C4—C5—N1—C9	−89.9 (3)
C3—C4—C5—C6	157.1 (2)	C6—C5—N1—C9	33.7 (3)
S1—C4—C5—C6	−28.1 (3)	N1—C9—N2—C7	−10.0 (4)
N2—C7—C6—C10	−177.2 (3)	S2—C9—N2—C7	167.7 (2)
C8—C7—C6—C10	3.3 (5)	C6—C7—N2—C9	14.3 (4)
N2—C7—C6—C5	7.0 (4)	C8—C7—N2—C9	−166.2 (3)
C8—C7—C6—C5	−172.5 (3)	O1—C10—O2—C11	−3.4 (6)
N1—C5—C6—C7	−27.6 (4)	C6—C10—O2—C11	178.7 (4)
C4—C5—C6—C7	95.4 (3)	C12—C11—O2—C10	176.3 (5)
N1—C5—C6—C10	156.1 (2)	C2—C1—S1—C4	−0.5 (3)
C4—C5—C6—C10	−80.9 (3)	C3—C4—S1—C1	1.1 (2)
C7—C6—C10—O1	169.5 (3)	C5—C4—S1—C1	−174.5 (2)

### Hydrogen-bond geometry (Å, º)

Cg1 is the centroid of the S1/C1–C4 thiophene ring.

**Table:**

*D*—H···*A*	*D*—H	H···*A*	*D*···*A*	*D*—H···*A*
N1—H1*N*1···S2^i^	0.86	2.63	3.408 (2)	151
N2—H2*N*2···O1^ii^	0.86	2.15	2.984 (3)	162
C12—H12*B*···*Cg*1^iii^	0.96	2.81	3.664 (6)	149

Symmetry codes: (i) −*x*+2, −*y*, −*z*+1; (ii) *x*+1, *y*, *z*; (iii) −*x*+1, −*y*+1, −*z*.
