# Supplementary figures and images for: Crystal structure of ethyl 6-methyl-2-sulfanyl­idene-4-(thio­phen-2-yl)-1,2,3,4-tetra­hydro­pyrimidine-5-carboxyl­ate
Source: Acta Crystallogr E Crystallogr Commun. 2015 Jan 3;71(Pt 2):o81–2. doi: 10.1107/S2056989014027741 (PMC4384618; doi:10.1107/S2056989014027741)

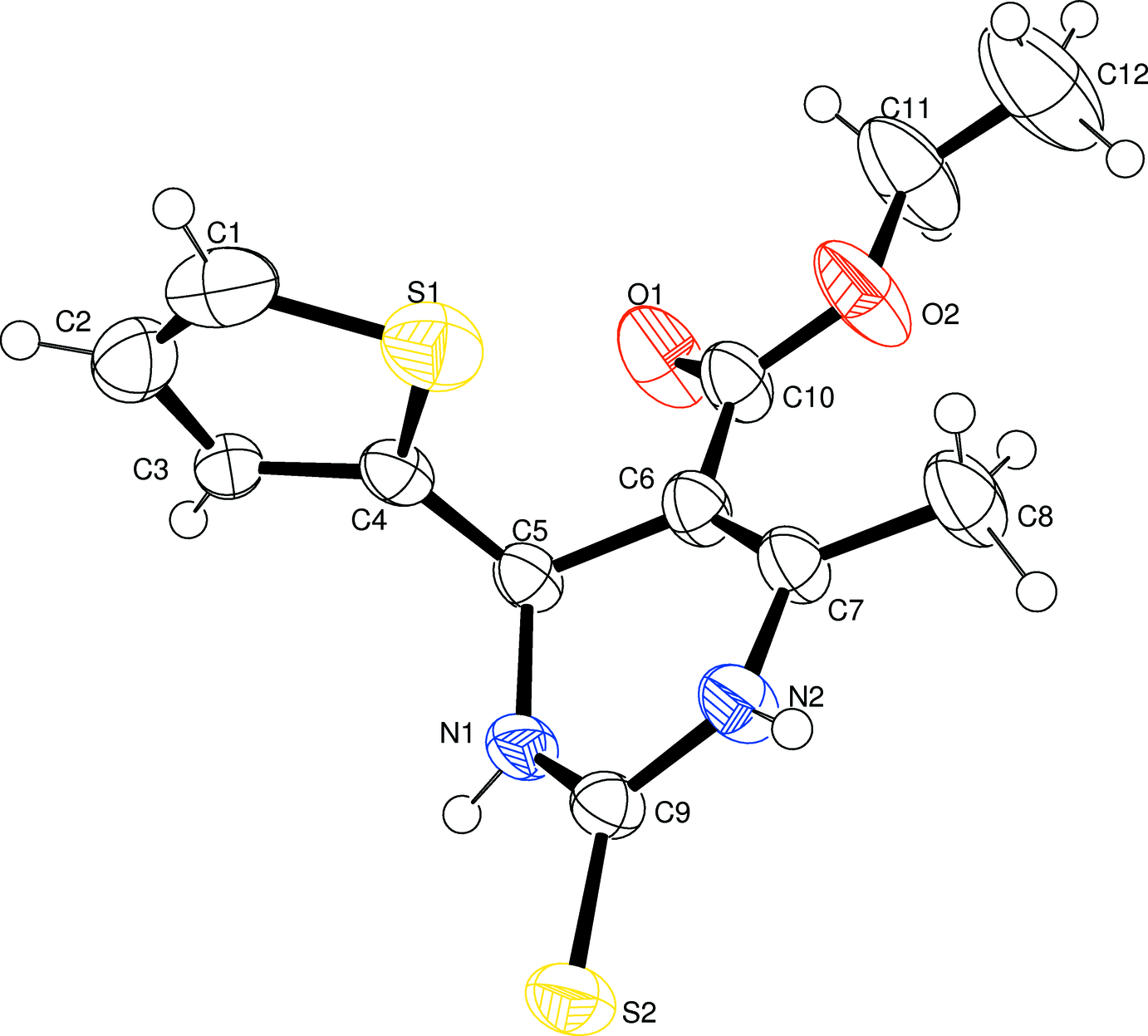

Supplement: Supplementary file 4 [file e-71-00o81-fig1.tif]

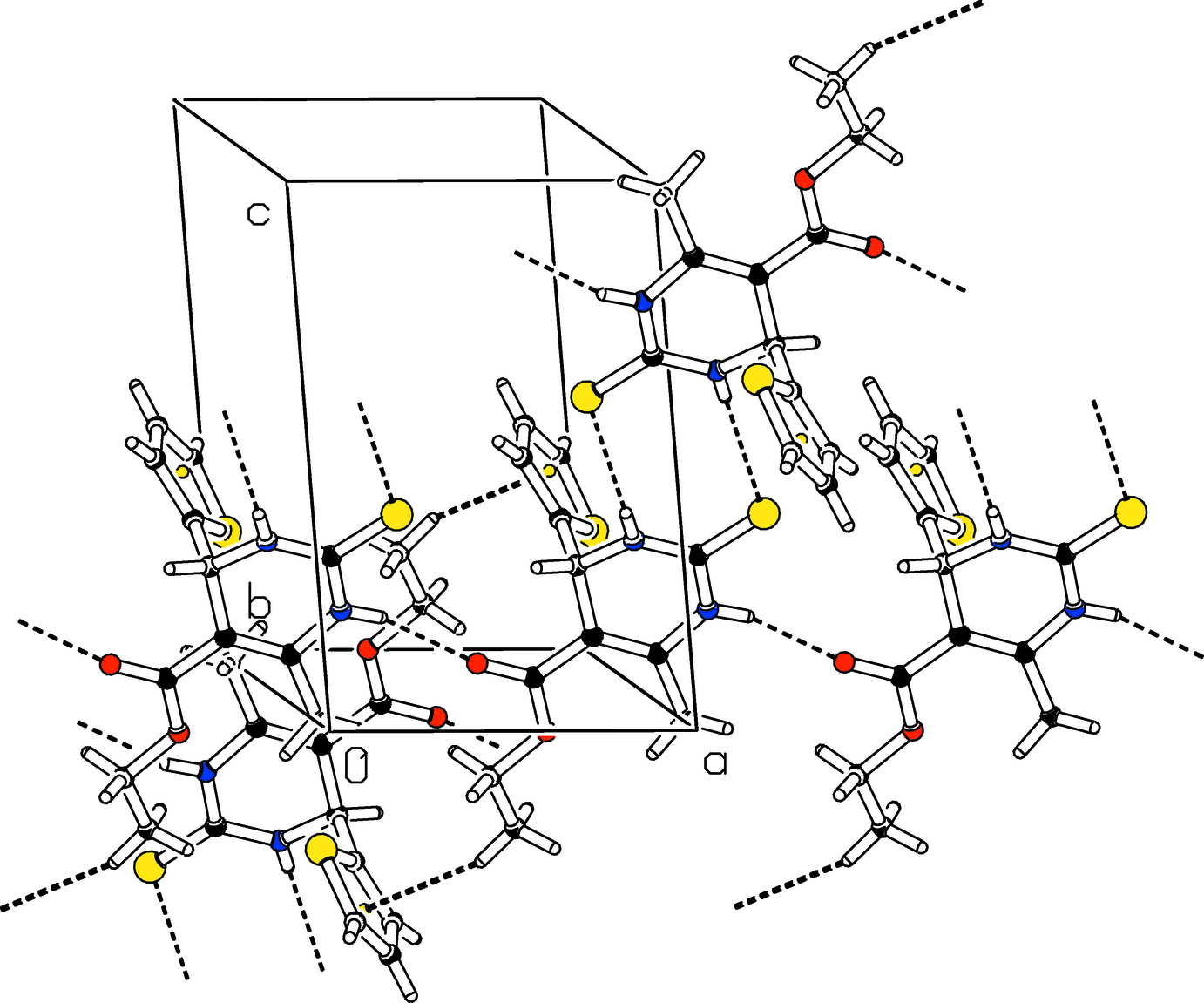

Supplement: Supplementary file 5 [file e-71-00o81-fig2.tif]

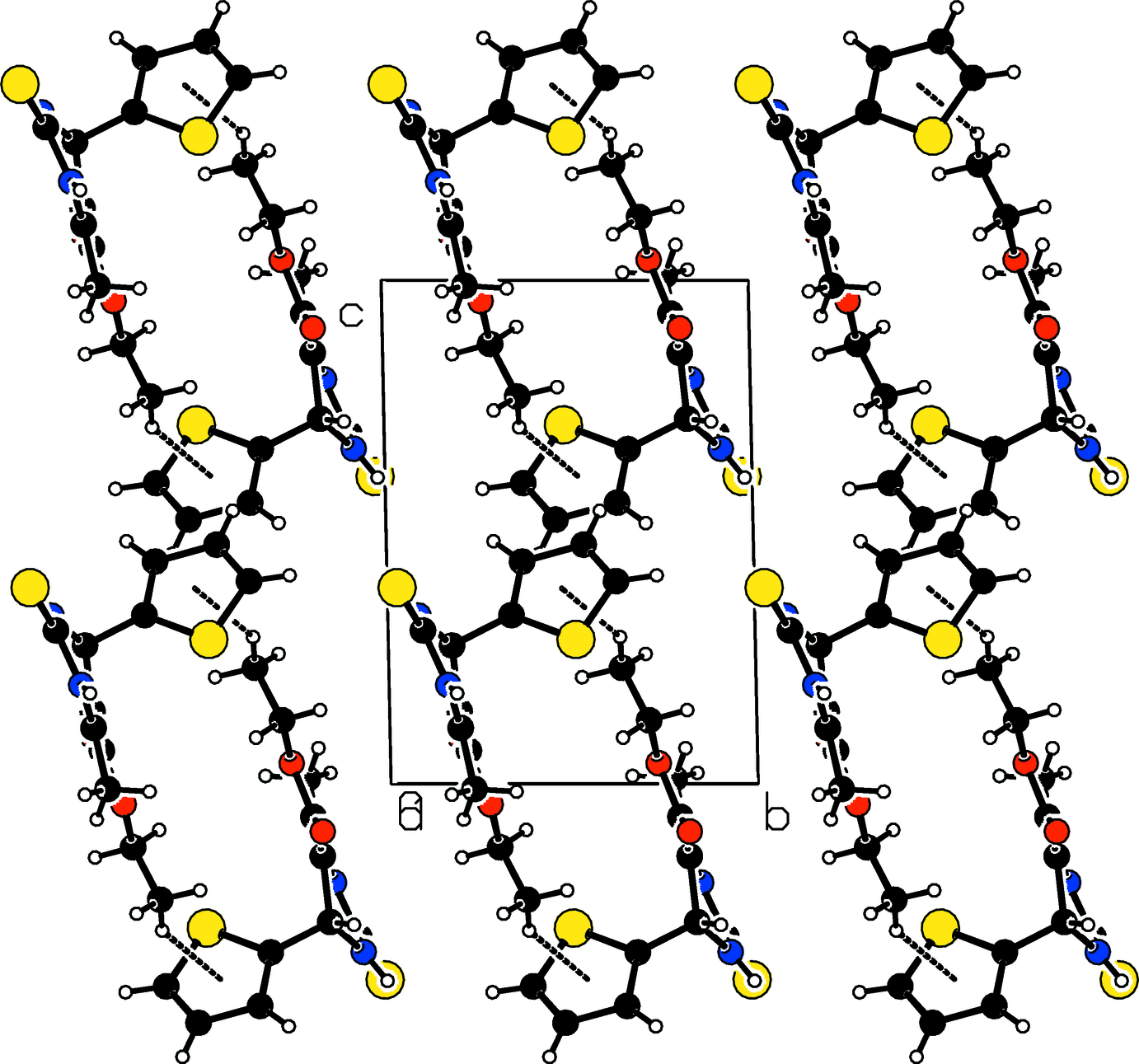

Supplement: Supplementary file 6 [file e-71-00o81-fig3.tif]

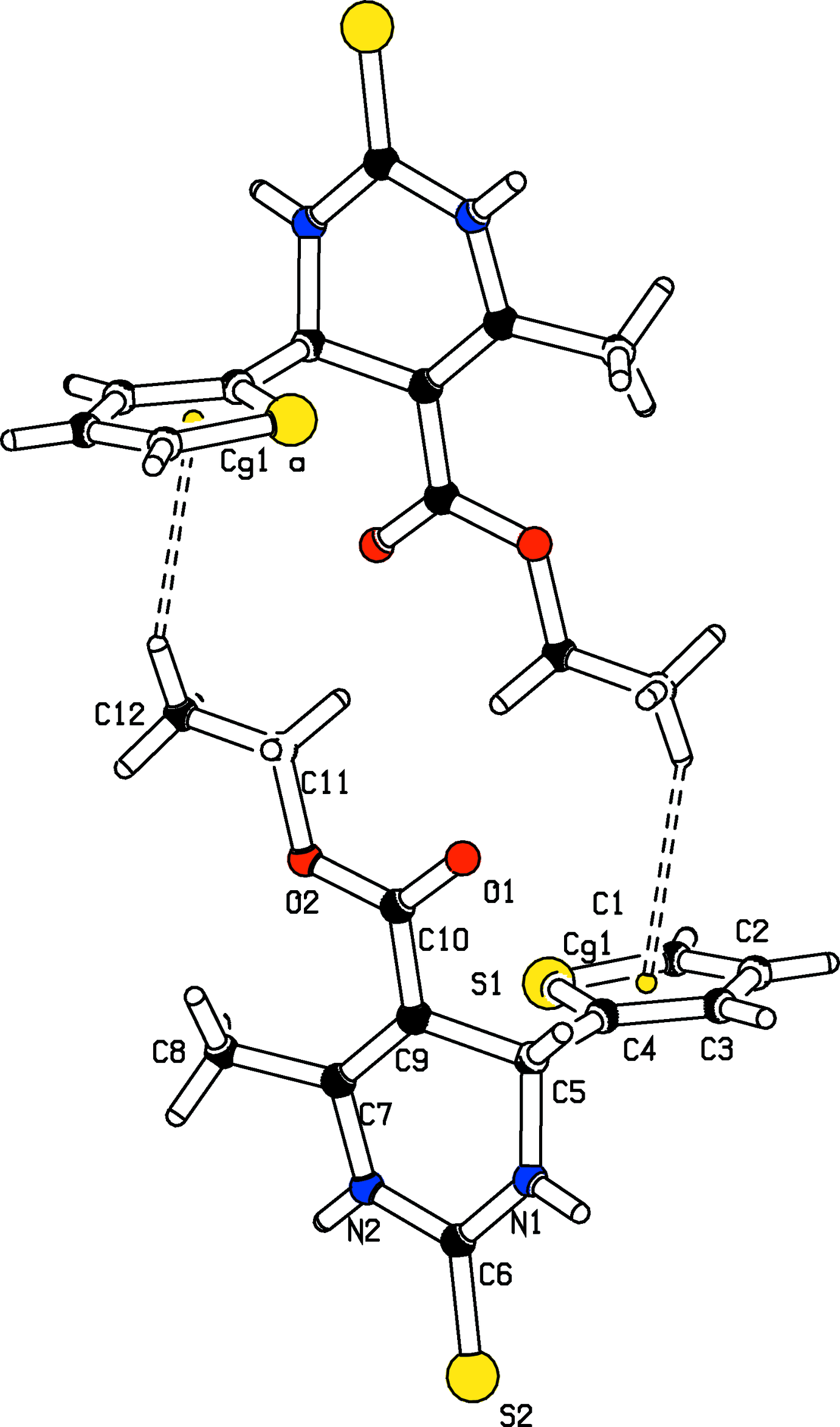

Supplement: Supplementary file 7 [file e-71-00o81-fig4.tif]
